# Normative 3D opto-electronic stereo-photogrammetric sagittal alignment parameters in a young healthy adult population

**DOI:** 10.1371/journal.pone.0203679

**Published:** 2018-09-07

**Authors:** Edyta Kinel, Moreno D’Amico, Piero Roncoletta

**Affiliations:** 1 Department of Rheumatology and Rehabilitation, Clinic of Rehabilitation, University of Medical Sciences, Poznan, Poland; 2 SMART Lab (Skeleton Movement Analysis & Advanced Rehabilitation Technologies) Bioengineering & Biomedicine Company Srl, Pescara, Italy; 3 Università degli studi G. D'Annunzio, Department of Imaging Neuroscience and Clinical Science, Chieti, Italy; Medical College of Wisconsin, UNITED STATES

## Abstract

This paper describes and presents a stable and reliable set of stereo-photogrammetric normative data for global and spino-pelvic sagittal alignment, as a proven reference system for evaluating/measuring a fully unconstrained natural upright neutral standing attitude in a young healthy adult population. The methodological features described in this article will enable future studies to replicate and/or directly compare a wide range of different postural tests and/or sagittal alignment assessment procedures including the study of sagittal spine shape variations occurring during gait performance. To date, the quantitative evaluation of adult spinal deformity (ASD) has been mainly confined to the X-ray imaging approach and, more recently, to 3D X-ray reconstruction. Within the existing evaluation framework an opportunity exists for an additional approach: a quantitative evaluation procedure which is easy, accurate, relatively speedy and non-ionising, in order to monitor and track the progress of patients in the areas of both surgical and non-surgical treatment. The resources and methodology described in this paper have been proven to meet all these criteria. They have enabled full 3D posture (including 3D spine shape and sagittal alignment of the skeleton) to be consistently and successfully measured in adult volunteers. All the measurement/evaluation procedures and outcomes carried out were based entirely on the new non-ionising 3D opto-electronic stereo-photogrammetric approach described in this article. The protocol for this methodology was based on a standard set of 27 pre-selected anatomical “landmarks” on the human body, providing standard reference points for observation and measurement. A total of 124 healthy subjects were successfully assessed and, for each subject, 27 individual markers were applied to the corresponding locations on his/her body. Statistical tests to investigate gender differences were also carried out. Descriptive statistics are provided for all 15 of the spino-pelvic parameters under consideration. Results indicated significant differences between genders in five sets of parameters: Kyphosis tilt, Head tilt, Pelvic tilt, Spino-pelvic angle and T1-pelvic angle. The data also demonstrate a high degree of congruity with results obtained using the X-ray method, as evidenced by the existing literature in the field. In summary, the current study presents a new stereo-photogrammetric opto-electronic technology which can be used successfully for ASD evaluation and introduces a comprehensive set of normative data analogous to those proposed in X-ray analysis for sagittal spino-pelvic and total body alignment.

## Introduction

Given its high prevalence in the adult population [1, 2] and the increasing social burden it creates [3–5] adult spinal deformity (ASD) is receiving ever-growing attention in both clinical and research fields for its potential economic impact in health care [2]. Currently, the prevalence of ASD in the context of scoliotic deformities is estimated to vary within a range of 1% to 30% of the adult population [6–8] and possibly rising as high as 68% in that portion of the population over 65 years of age [6].

With regard to hyperkyphosis specifically, its prevalence in older individuals is not precisely known but has been reported to be between 20% and 40% [9, 10]. This high level of prevalence might be considered within the context of an ageing population in Western countries generally. For example, in the European Union (28 countries, 511.5 million people in 2017), the older persons, aged 65 or over, had a 19.4% share, according to statistics provided by EUROSTAT [11]. Moreover, the problems associated with ASD are not confined to the field of Orthopaedics and there are indications a “vicious cycle” could develop more broadly: Bess et al [12] demonstrated that the mental and physical disability caused by spinal deformity was comparable to that caused by cancer or diabetes.

Recent research and clinical work addressing basic issues of diagnosis and treatment of ASD is placing increasing, and more focused, attention on features such as sagittal plane global posture, spine shape, and spino-pelvic balance. Roussouly and Nnadi [3] have demonstrated the importance of accurate, comprehensive information on the position of the lower extremities as well as the pelvis in the context of any spinal pathology. It has been established that spinal deformation, which can be local, regional or global [13] can also have an indirect impact on the hip [4,3,14], the lower limbs [3,15] and the soft tissues. Using health-related quality of life (HRQOL) measures and radiographic analysis, research has also been able to demonstrate the relationship between pain and disability experienced by patients and a corresponding deterioration in sagittal alignment [4, 5]. It is therefore logical and appropriate that the preservation and/or restoration of global sagittal alignment (a relatively complex issue) are key objectives of both conservative and more invasive surgical treatments [3,14,16,17]; this more complex approach is superior to a relatively limited focus on the spine alone.

At this point in time, quantitative evaluation of ASD has largely been confined to the X-ray imaging approach. This approach embodies ever-increasing technological developments, applying techniques of planar X-ray analysis to 3D reconstruction [18–20]. However, although indispensable, the radiological approach to evaluation of global skeleton posture might also be limiting in ways that could hinder full functional assessment and, most importantly, might not be considered optimal for continuous monitoring of the patient’s status. In this context, the opto-electronic stereo-photogrammetric measurement system as described in this paper offers a helpful and meaningful solution for the capture of functional information necessary for addressing clinical problems [21]. A more extensive review of the non-ionising methods presented in the literature is beyond the limit of the present paper; interested readers can find a thorough review of such an approach in D’Amico et al. [21].

In this paper we focus on reconstruction of the full 3D skeleton using the stereo-photogrammetric approach originally introduced by D’Amico et al. in 1995 [22]. The fully-developed final version of that approach was presented and described in a very recent paper [21]. In the same recent paper, a comprehensive set of normative data in a young healthy adult population was introduced. The present research aims to complement this existing set of normative data by introducing new additional parameters for sagittal spino-pelvic and total body alignment, analogous to those proposed in the X-ray analysis literature [3,18]. This extended research was designed to create a broader reference set for our non-ionising quantitative approach, thus enabling further study in the field of postural modification at all stages of life from childhood to old age.

## Materials and methods

**Fig 1:**
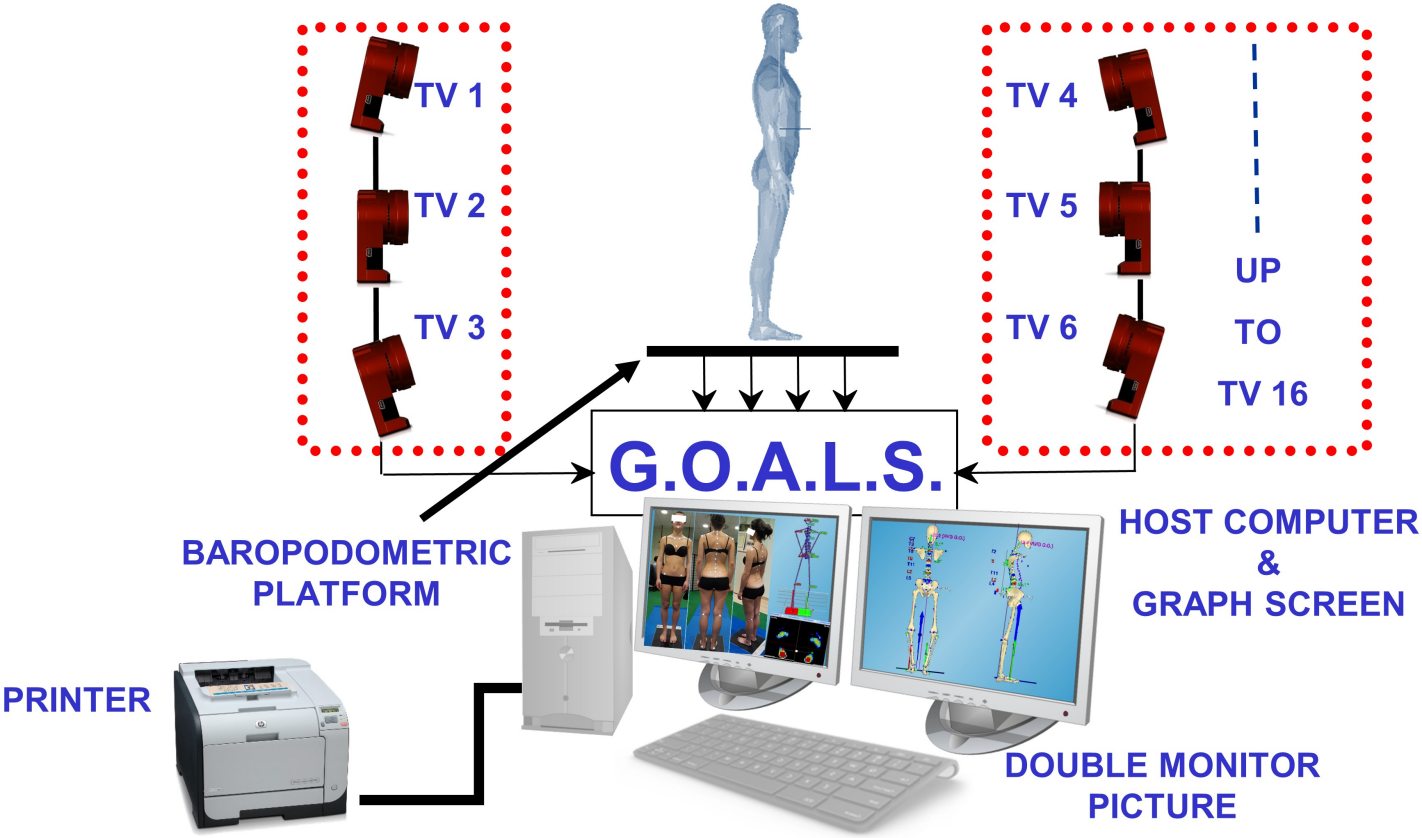
GOALS system: Basic configuration.

**Fig 2:**
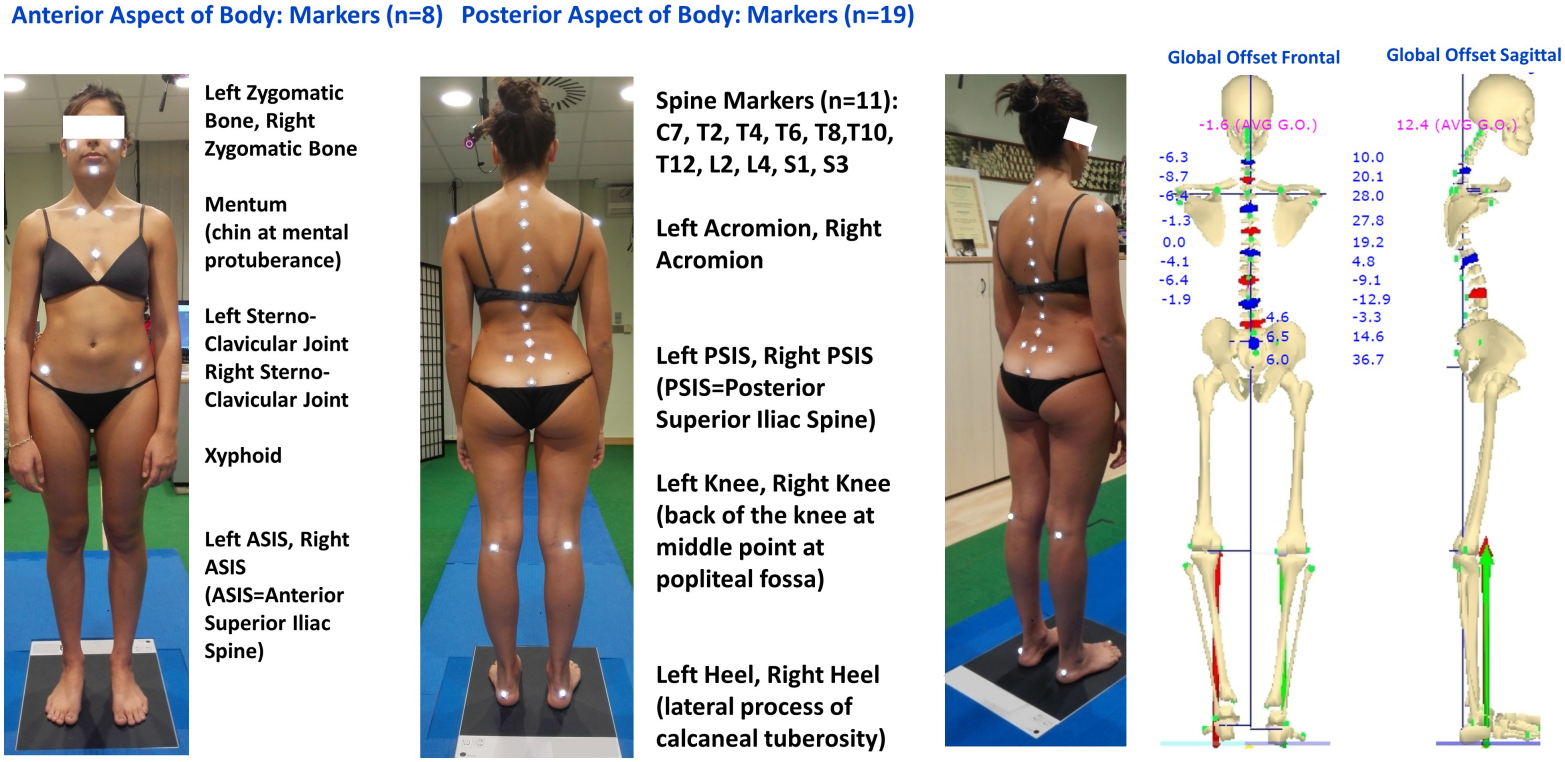
27 markers set for full skeleton 3D posture measurement.

Our experimental recordings are based on a new 6TVC (resolution 1.3 Mpix, 120fps) G.O.A.L.S. (Global Opto-electronic Approach for Locomotion & Spine) (Bioengineering & Biomedicine Company S.r.l. Italy) stereo-photogrammetric opto-electronic system derived from Optitrack System (NaturalPoint Inc. USA), Fig 1. Data processing was carried out using a specially developed software package named ASAP 3D Skeleton Model (Bioengineering & Biomedicine Company S.r.l. Italy). In this elaboration software, a complete 3D parametric biomechanical human skeleton (3D spine included) has been modelled mathematically; the skeleton can be accurately scaled by fitting the 3D anthropometric bone size to the corresponding 3D opto-electronic measurements of a series of suitable body landmarks. After calibration the GOALS system provided 0.3–0.4mm 3D accuracy in 3D marker position reconstruction on a 3m x 3m x 2m working volume used for the present study. To analyse human posture and spinal related pathologies, a protocol of 27 body landmarks labelled by passive markers (Fig 2) was established and tested extensively in the clinical environment [21–25]. This protocol, named “ASAP POSTURE”, has been deposited under that name in *protocols*.*io* and has also been assigned its own identifier (DOI) [26]. This general methodology can be applied indifferently to any stereo-photogrammetric recording system, provided that the latter is able to accommodate all the required landmarks as three-dimensional coordinates. Hemispheric (10mm diameter) retro-reflective passive markers were selected for use on the spine and pelvis in order to minimise the bone-prominence/marker distance and reduce geometrical interferences [22,27]. Moreover, for all subjects, marker positioning was determined by palpation by a single operator with more than 20 years experience, while the subject was standing.

In our data we do not include an explicit comparison of this method with X-ray measurement: this paper does not aim to validate the method under consideration. Validation and discussion of this method have been extensively treated in previous papers by D’Amico et al. [21–25]. Conversely, sagittal spino-pelvic and total body alignment parameters from the X-ray based literature will serve as the “gold standard” to establish the degree of disparity/congruity between the well-known and predominant X-ray approach on one hand, and the 3D stereo-photogrammetric based measurement approach described in this paper.

## Design

The research presented here is an observational cross-sectional study. A cohort of 124 asymptomatic young adult university students and workers was measured to establish the baseline normative data. All participants were volunteers: a signed consent form was required and provided in every case.

### The study population

All subjects in the cohort that formed the study population shared the following characteristics: Caucasian males or females in the age range 19–35 years; all subjects declared that they undertook no sports activity at an agonistic level, had no orthopaedic or neurologic problems and no history of musculoskeletal system injury or surgery. In all cases the Body Mass Index (BMI) of the subjects fell within normal range: 18.50 = < BMI = < 24.99, as established by the World Health Organisation (WHO) [28]. All subjects in this research participated in a face-to-face interview and a thorough clinical postural examination before their session of postural measurement. To ensure consistency, all the interviews and physical examinations were conducted by a single qualified physiotherapist with 15 years experience (the first named author) in order to confirm the subjects were free from any orthopaedic/neurological disorder. In case of any doubt, the researchers relied upon the intervention of one or more physicians from the Local Medical Committee to provide a professional medical opinion.

### Compliance with ethical standards

The study was carried out in conformity with the Declaration of Helsinki and—given the innocuous quality of the measurement process, the focus on merely postural characteristics of healthy subjects, strict data privacy protection (data are published anonymously and only in statistical form) and the voluntary participation of all the subjects—the research was formally approved by the Local Medical Ethics Committee and confirmed by Bioethics Committee, Poznan University of Medical Sciences (n. 1255/16). After being fully informed about the risk-free nature of the evaluation and the aims of the research, each subject voluntarily signed a consent form.

Population characteristics are summarized in Table 1. The study was carried out and described according to the STROBE guidelines for reports describing observational studies [29].

**Table 1:** Sample population characteristics: Total 124 healthy young adults.

Population Characteristics	Female (n = 57)		Male (n = 67)		t-Test Female vs Male
	Range	Mean± SD^a^	Range	Mean± SD^a^	
**Age [years]**	19–34	23.5±3.2	20–35	24.9±3.9	NS^b^
**Height [cm]**	155–175	163.9±5.3	164–190	178.3±6.7	P<0.001
**Weight [kg]**	40–71	56.1±7.0	50–90	71.8±8.6	P<0.001
**BMI [kg/m**^**2**^**]**	15.6–24.8	20.8±2.0	18.6–24.9	22.5±1.6	NS^b^

^a^SD = Standard Deviation

^b^NS = Not Significant

### Acquisition protocol

The assessment/measurement session aimed to fully capture and record the subject’s neutral unconstrained erect-standing posture. The subject was asked to align his/her heels on a line parallel to the frontal plane and to keep feet apart (with no guidance as to direction or alignment of the feet) at about pelvis width (i.e. with feet under the projection of the hip joints); in addition, subjects were asked to keep the upper arms relaxed along the side of the body, eyes looking directly ahead in the horizontal plane and mouth closed. The desired static postural attitude was considered to have been accurately recorded when at least five acquisitions, each of two seconds duration, were captured by the opto-electronic device and successively averaged. Given the 120Hz opto-electronic device data acquisition rate, this means that a minimum of 1200 measurements was averaged per each static postural stance [21–25]. Furthermore, in order to reduce potential postural effects resulting from circadian rhythms, all measurements were taken between 12 noon and 7:00 pm. The subjects were asked to avoid any intensive training and/or hard physical activity before the postural assessment.

### Sagittal alignment parameters

**Fig 3:**
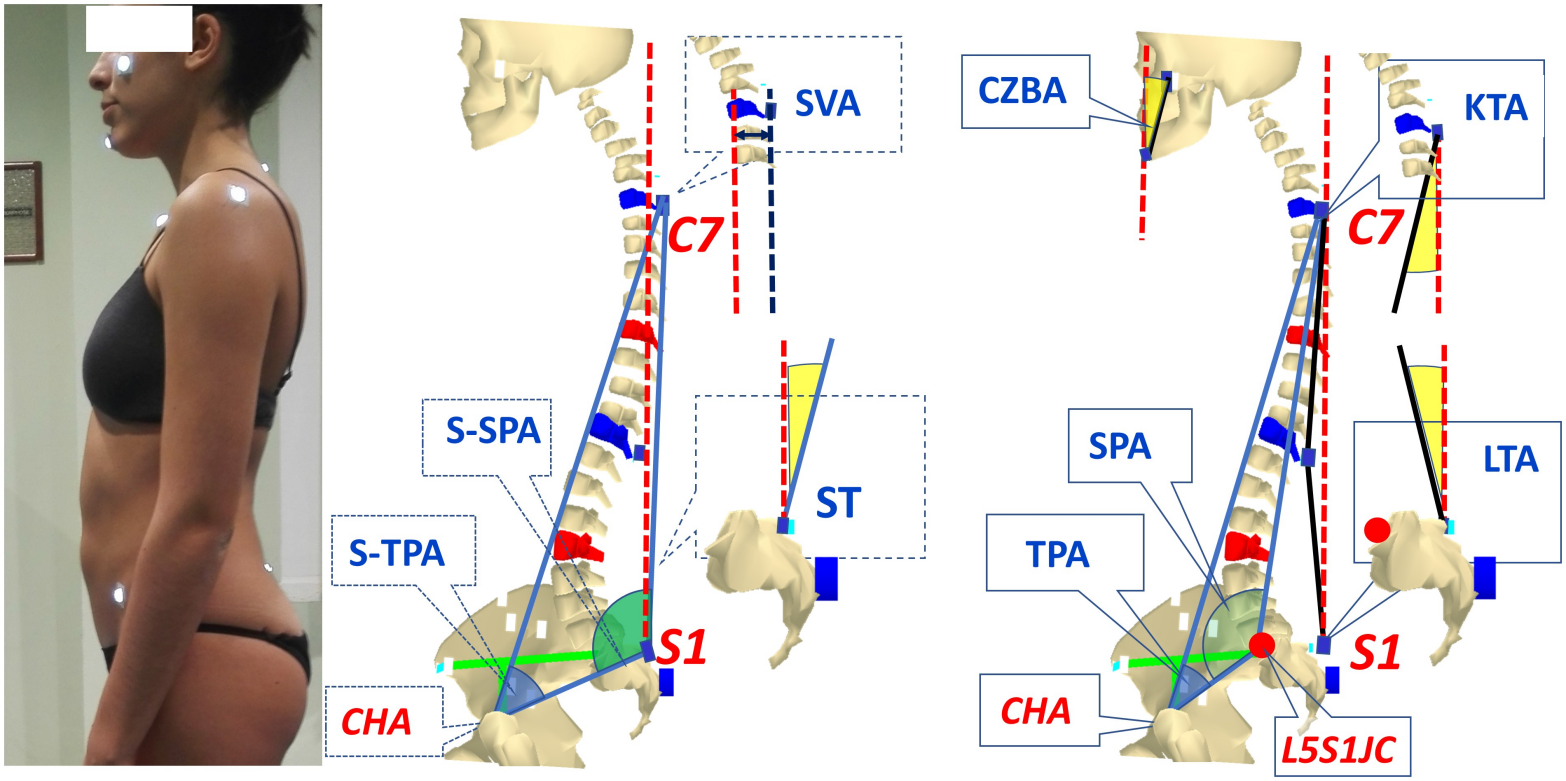
Spino-pelvic parameters. See definitions in Table 2.

**Fig 4:**
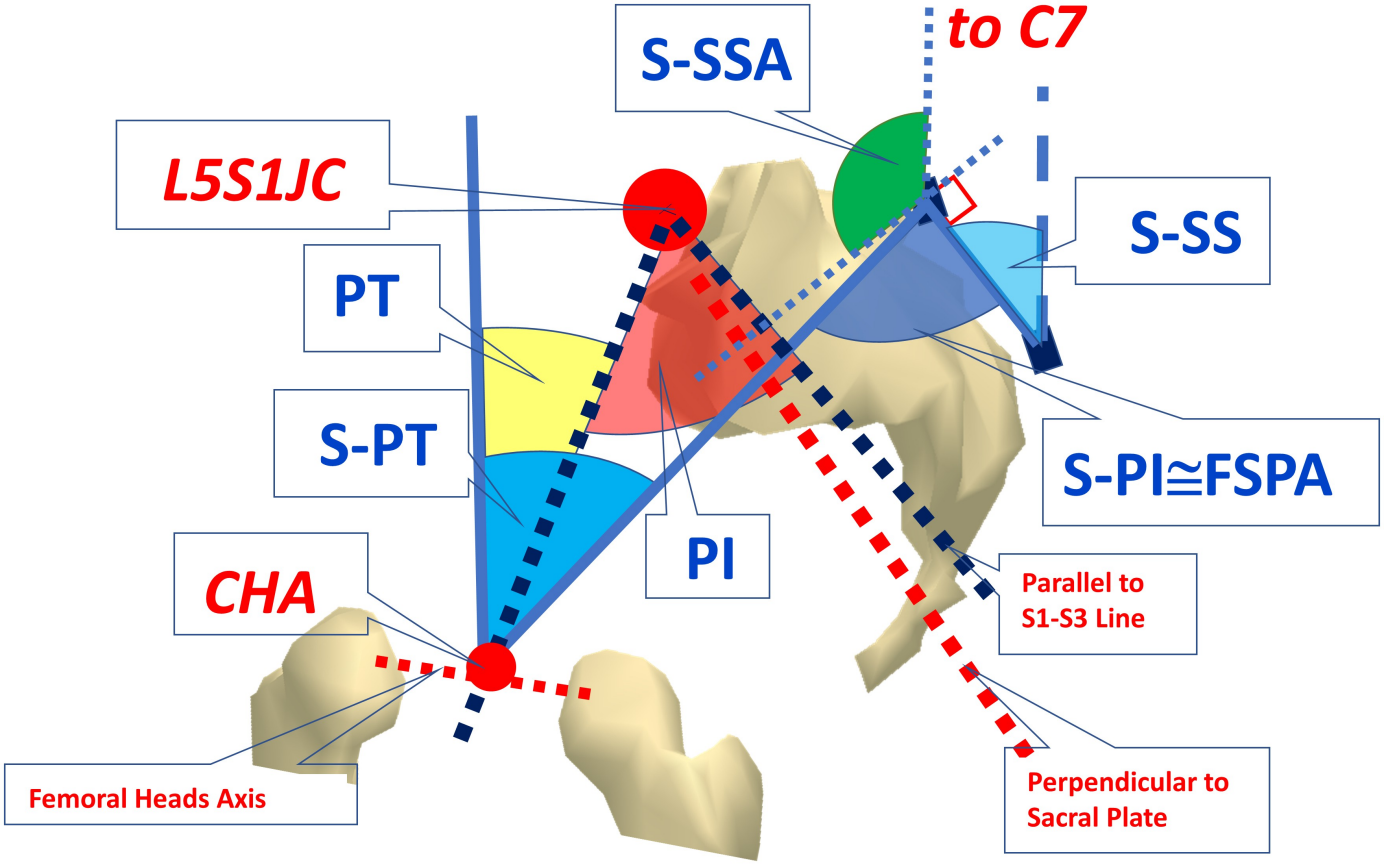
Pelvic parameters. See definitions in Table 2.

In this paper we introduce a new set of parameters analogous to those used in X-ray analysis for sagittal spino-pelvic and total body alignment. In particular, we chose a selection from those proposed in a paper by Roussouly and Nnadi [3] and from the review carried out by Vrtovec et al. [18]. Some of these computed parameters, as they were originally obtained using the processes of X-ray analysis, cannot be directly derived using the stereo-photogrammetric approach. In these cases, similar or related parameters were substituted. The list and definition of the selected parameters are given in Table 2, and graphical representation of them is given in Figs 3 and 4.

**Table 2:** List and definition of quantitative biomechanical parameters considered by this research; and related abbreviations (n = 15).

Abbreviation	Description	Definition
**CZBA**	Chin Zygomatic Bones Angle^a^	The CZBA is computed as the angle in the sagittal plane between the vertical axis and the line joining the middle point between left and right zygomatic bones and the chin. This represents the stereo-photogrammetric estimate of the Chin-Brow vertical angle computed on radiographs [3].
**KTA**	Kyphosis Tilt Angle^a^	The kyphosis tilt angle is the angle between the vertical axis and a line drawn from the superior to the inferior kyphosis limit vertebrae [3]. This angle describes the tilt induced by global kyphosis. It is important to note that in our approach the limit-vertebrae (i.e. vertebrae marking the beginning and the end of each identified curve) are defined as curve inflection points (see text for explanation).
**LTA**	Lordosis Tilt Angle^a^	The lordosis tilt angle is the angle between the vertical axis and a line drawn from the superior to the inferior lordosis limit vertebrae [30].
**ST**	Spinal Tilt^a^	This is the angle between the vertical axis and a line drawn from C7 to S1 [3].
**PT**	Pelvic Tilt	This is the angle between a vertical line originating at CHA and a line starting from CHA to L5S1JC. In simple terms, this angle describes the rotation of the sacral endplate to the hip axis [3].
**S-PT≅PA**	Stereo-Photogrammetric Pelvic Tilt≅PA	This is the stereo-photogrammetric version of PT angle between a vertical line originating at CHA and the CHA-S1 line. This angle as defined is very close to the pelvic angle PA described in Vrtovec et al. [18]
**S-SS**	Stereo-Photogrammetric Sacral slope	This is the angle between a vertical line originating at S3 vertebra and the line joining the S1 and S3 vertebrae. In this paper, we use the perpendicular to the S1-S3 line to assess the X-ray Sacral Slope [18].
**PI**	Pelvic Incidence	In an X-ray image, this is the angle subtended by the perpendicular to the sacral plate at its mid-point and a line from the mid-point of the sacral plate to the centre of the femoral head [3]. In our stereo-photogrammetric approach, using the definition given for the S-SS parameter, we compute the angle between the line originating at CHA to L5S1JC and a line starting from L5S1JC parallel to S1-S3 line.
**S-PI≅FSPA**	Stereo-Photogrammetric Pelvic Incidence	This is the angle between the line originating at CHA to the S1 spinous process and the S1-S3 line. This angle as defined is very close to the femorosacral posterior angle FSPA described in Vrtovec et al. [18].
**SPA**	Spinal Pelvic AngleL5-S1	In an X-ray image, this is the angle between a line from the centre of C7 to the centre of the sacral endplate and a line from the centre of the sacral endplate to the centre of the femoral head [3]. In our stereo-photogrammetric approach, we compute the angle between the line originating at C7 to L5S1JC and a line from L5S1JC to CHA.
**S-SPA**	Stereo-Photogrammetric Spinal Pelvic Angle	This the angle between the line C7-S1 and the line S1-CHA.
**S-SSA**	SpinoSacral Angle	In an X-ray image, this is the angle between a line from the centre of C7 to the centre of the sacral endplate and the surface of the sacral endplate [3]. In our stereo-photogrammetric approach, using the definition for the S-SS parameter, we compute the angle between the line originating at C7 to L5S1JC and a line perpendicular to S1-S3 from L5S1JC.
**TPA**	T1 Pelvic Angle	In an X-ray image, the T1 pelvic angle is defined as the angle between the line from the assessed centre of femoral heads axis to the centroid of T1 and the line from the femoral heads axis to the middle of the S1 superior endplate [31]. In our stereo-photogrammetric approach, we compute the angle between the line originating at C7 to CHA and the line from CHA to L5S1JC.
**S-TPA**	Stereo-Photogrammetric T1 Pelvic Angle	This is the angle between the line originating at C7 to CHA and the line CHA-S1.
**SVA**	Sagittal Vertical Axis	This is the distance computed from S1 to a plumb line dropped from C7 [3]. A value >0 means that the C7 is anterior to S1.

^a^ When the value of the computed angle is >0 the tilt is forward leaning

In Table 2 and associated Figures the following definitions are applied:

- In an X-ray-based image analysis, the computed lines for postural alignment parameter extraction usually originate and end at the centre of a vertebral body; or at the centre or a corner of its superior endplate. In our stereo-photogrammetric approach, the lines are always drawn between the markers placed on spinous processes.
- The computation of some parameters requires the identification of the centre of the hip axis (CHA), i.e. the middle point of the segment joining the hip joint centres. These latter are assessed based on measurements of Anterior and Posterior Iliac Spine positions (ASIS and PSIS) by using regression equations [32].
- The following X-ray based parameters: PI, PT, SPA, SS, SSA, TPA, FSPA (Table 2) are computed for spino-pelvic alignment, using S1 vertebral plate slope, S1 plate centre or finally S1 posterior corner spatial position, but they cannot be acquired directly from skin marker measurements. For this reason, to compute spino-pelvic parameters using the stereo-photogrammetric approach, the perpendicular to the line passing through S1-S3 markers has been used to assess the sacral slope plate (S-SS). Furthermore, we used the two following different approaches:
  - In addition to the assessed position of CHA [32], an estimate of the L5-S1 joint centre (L5S1JC) location also derived from PSIS and ASIS locations [33,34] has been utilised in order to remain as close as possible to the X-ray-based definition. The following parameters have been extracted using this approach: PT, PI, SPA, TPA (Table 2).
  - As the approach above is strongly dependent on the reliability of regression equations presented in the literature, we introduced a second approach so as to reduce the possible effects of any such variance. In this case, the L5S1JC assessment was replaced by the position of the marker placed on the S1 spinous process. We defined the following parameters in this way: S-PT, S-PI, S-SPA, S-SSA, S-TPA (Table 2).

The main features of the procedure used by the authors to determine the key elements of the relevant measurement parameters are set out below. The full analytical and mathematical descriptions of the entire procedure to extract the 3D spine shape and 3D spino-pelvic parameters are beyond the aim of this paper and can be found in the research papers as cited [21–24,35–39]. In this paper only the main features will be described.

#### Summary of analytical and mathematical procedure for spino-pelvic parameters computation

First, and before averaging, a degree of pre-processing of the acquired 3D raw data is necessary in order to comply with clinical analysis requirements [21]. To begin, the subject's local coordinate system must be defined; for this we have used the general definitions provided by the Scoliosis Research Society [40]. Thus, in a right-handed system: the frontal-coronal (YZ) plane is the vertical plane containing the PSIS (where the Y-axis is the horizontal medio-lateral axis belonging to this plane) starting from the projection of the S1 landmark on the frontal-coronal plane—defined as the origin of the subject’s local reference system—pointing from the right to the left of the body. Z is the vertical axis pointing upwards from the origin of the system; the sagittal plane is vertical and orthogonal to the frontal-coronal plane passing through the origin of the system (the X axis is orthogonal to the previous Y and Z axes pointing forward relative to the body and defining the Anterior-Posterior direction); the transverse-horizontal plane is orthogonal to both the previous two planes passing through the origin of the subject’s reference system [21].

Having determined this individual system, a rotation is then performed within each frame in order to align the subject’s coordinates with the global reference coordinates. Once the alignment is complete, it is possible to proceed to a proper averaging of all acquired frames. Based on measurements of the eleven 3D spinous processes, noisy data are interpolated using cubic splines [41] in order to assess the position of each unlabelled spinous process and intervertebral disc. This allows us to capture anatomical structure and location with strict accuracy relative to the mathematical-geometrical representation. After interpolation, the space-curve modelling of the spine is analytically represented by means of three parametric functions x(t), y(t), z(t), the parameter being t > 0. A specially developed smoothing and differentiation procedure for cubic spline interpolated data is applied to these functions [22,35–37,42]. The results of filtering signal processing are then used to provide inputs for the automated procedure of identifying the limit-vertebrae (ie, vertebrae marking the beginning and the end of each identified curve in both planes) defined as curve inflection points. In particular, the frontal and sagittal spine shapes are derived from the filtered 3D analytical representation of the spine by mathematical projection onto the respective planes. Subsequently frontal and sagittal spine shape curves are processed separately and the first and second derivative functions are assessed. The analysis extends from the upper labelled C7 down to the S3 spinous processes: thus, the inflection points are selected under analytical constraint, and in fact these are the points at which the second derivative of the two average curves crosses the zero line [43]. In this way kyphosis (ie, backward-facing convex curve) and lordosis (ie, forward-facing convex curve) are properly identified according to real spine curvature spatial changes at the limit-vertebrae: they are no longer restricted to specific thoracic or lumbar anatomical regions. This enables kyphosis to be identified as longer or shorter than the thoracic anatomical region and, in the same manner, lordosis can extend from the lower thoracic level to the sacral region [21].

### Statistical analysis

The statistical analysis has been managed at various levels. The data have been analysed using the Real Statistics Resource Pack software (Release 4.7) [44] with a significance level of α < 0.05.

#### Descriptive statistics

Descriptive statistics were extracted for all parameters in question and, when a statistically significant difference was found between genders, for males and females separately.

#### Group statistical analysis

The subject cohort was subdivided into two groups, male and female. The minimum sample size was calculated *a priori* and based on an 80% probability of detecting a mean difference between the two groups equivalent to 1 SD (standardised effect size d = 1). The minimum number of male and female subjects necessary for this research project to be meaningful, based on the analytical criteria above, would need to be 17 individuals per group. The recruitment campaign managed to attract a greater number of subjects, for a total of 57 females and 67 males as described in Table 1. Given that the final number of available participants was more than sufficient, postural characteristics for both genders were compared for each variable of interest, applying a set of unpaired t-Tests (leading to a standardised effect size d = 0.508, power = 80%) or, where assumptions for normality and/or homogeneity of variances were not satisfied, the Mann-Whitney non-parametric equivalent tests [44, 45].

#### Type classification vs. gender statistical relationship

The process of classification of the normal variation in the sagittal alignment in the standing position, identifying four different patterns (analysing the length ratio of the thoracic and lumbar curves) was first introduced in a paper by Roussouly et al. [30].

In this present research project the authors also investigated whether any difference could be detected between genders with regard to classification patterns: ie, rates of occurrence for each gender within each pattern. The Chi-Square Test of Independence was selected as the most appropriate tool for this aspect of the research.

## Results

### Descriptive statistics—Group statistical analysis

The Sagittal Alignment Normative Data extracted from the sample population are reported in Table 3. Those variables that produced a statistically significant difference between male and female groups are listed separately. Conversely, for variables that did not produce statistically significant differences between the sexes, the reported value was obtained by combining all males and females into a single sample. In this way the normative data for a total of fifteen sagittal spino-pelvic parameters was acquired.

**Table 3:** Sagittal alignment normative data and female vs male comparison.

3D Sagittal Alignment Spino-Pelvic Parameters	Females n. 57, Males n. 67, Total n. 124			
	Mean± SD	Ranges: min-max	t-Test^a^ or ^b^Mann-Whitney	Cohen Effect Size
**SVA [mm]**	22.2 ± 18.1	-32.6–72.0	Not Significant^a^	0.333
**KTA**_**Males**_ **[degree]**	4.4 ± 3.2	-4.8–11.5	P = 0.0126^a^	0.456
**KTA**_**Females**_ **[degree]**	2.7 ± 4.1	-7.– 12.8	P = 0.0126^a^	0.456
**LTA [degree]**	-0.9 ± 4.1	-9.5–10.9	Not Significant^a^	0.056
**ST [degree]**	2.7 ± 2.2	-4.0–8.2	Not Significant^a^	0.213
**CZBA**_**Males**_ **[degree]**	-17.2 ± 6.5	-12.6–17.7	P = 0.00023^a^	0.684
**CZBA**_**Females**_ **[degree]**	-12.6 ± 6.8	-14.5–9.0	P = 0.00023^a^	0.684
**S-PT [degree]**	49.6 ± 6.0	33.0–61.0	Not Significant^b^	0.205
**PT**_**Males**_ **[degree]**	17.3° ± 4.8°	4.7°– 30.1°	P = 0.038^a^	0.377
**PT**_**Females**_ **[degree]**	15.3 ± 5.7	0.7°– 29.6°	P = 0.038^a^	0.377
**S-SS [degree]**	16.5 ± 5.7	0.0–35.4	Not Significant^a^	0.239
**S-PI [degree]**	66.2 ± 6.3	51.4–85.0	Not Significant^a^	0.015
**PI [degree]**	33.2 ± 6.7	14.4–51.9	6.8±4.14^a^	0.012
**S-SPA [degree]**	127.5 ± 6.6	113.4 145.6	Not Significant^b^	0.258
**SPA**_**Males**_ **[degree]**	169.4 ± 5.6	152.1–179.4	P = 0.00325^b^	0.638
**SPA**_**Females**_ **[degree]**	173.0 ± 5.7	160.4–188.4	P = 0.00325^b^	0.638
**S-SSA [degree]**	103.7 ± 6.3	86.1–124.1	Not Significant^a^	0.292
**S-TPA**_**Males**_ **[degree]**	41.5 ± 4.9	31.2–50.9	P = 0.041^b^	0.438
**S-TPA**_**Females**_ **[degree]**	39.3 ± 5.4	26.6–49.0	P = 0.041^b^	0.438
**TPA**_**Males**_ **[degree]**	8.6 ± 4.5	0.5–21.7	P = 0.0028^b^	0.661
**TPA**_**Females**_ **[degree]**	5.6 ± 4.6	-6.6–15.6	P = 0.0028^b^	0.661

^a^ t-Tests

^b^ Mann-Whitney non-parametric equivalent tests, where assumptions for normality and/or homogeneity of variances were not satisfied.

### Type classification vs. gender statistical relationship

The distributions for classification types I, II, III and IV [30] were found to be 0.8%, 9.7%, 83.9% and 5.6% respectively. No significant differences were found between genders as regards the occurrence and frequency of each classification type.

## Discussion

Some of the stereo-photogrammetric parameters presented in this paper demonstrate measurement-values very similar to those reported in the literature for X-ray imaging analysis, for healthy populations of the same age range as those considered in the present study. (See also D’Amico et al. [21] for a thorough review of the X-ray based literature relative to spine shape parameters.) This should not be surprising, given that definitions for both systems are intrinsically geometrically equivalent, apart from the evident difference that for the stereo-photogrammetric system, measurements are taken from markers positioned on the skin above the spinous processes.

In particular, the definitions used for measuring and describing the SVA and the global or segmental spine tilts are inherently analogous. A wide range of SVA normative values can be found in the literature, spanning from +40mm±63mm [46] to -36mm±33mm [47] with a variety of intermediate values offered by a number of different authors [48–53]. The SVA as measured by the stereo-photogrammetric system (22.2mm ±18.1mm) sits squarely and entirely within the given range. One explanation for SVA normative values acquired from the X-ray system spanning such a broad spectrum of measures might be related to the postural attitude maintained by the subject during the measurement process. As reported in the literature, during X-ray investigations subjects assume a variety of different standing postures, to avoid possible occlusion of the spine in the lateral radiographs as a result of interference by the upper limbs.

Measurements are described as having been taken with the subject standing in a “comfortable” (non-specific) position with shoulders flexed 30°, 45°, up to 60° or even 90°. The position of arms and hands are described as: held in a raised position to reach the requested flexion angle at the shoulder; or resting on supports; or with fingertips placed on the mid clavicle; or finally with hands placed on the cheeks [54–56]. As has been clearly explained in the study by Marks et al. [48], standing lateral radiographic positioning does not represent customary standing balance. They found that when subjects stood with 45° of flexion of the shoulders and with upper limbs held in a raised position, a backward (negative) shift in SVA of about 6 cm was observed (with a range from 2.3 cm to 11.1 cm in all subjects) compared to a relaxed natural-neutral unconstrained erect-standing posture. Conversely, Vedantam et al [57] described that 35% of their patients demonstrated a forward (positive) shift in SVA with increased shoulder flexion from 30° to 90°; however in this study patients were positioned with their hands resting on a support. This indicated that the specific location and/or support of the hands induced a completely different effect in the degree of compensation required and generated by the spine and lower extremities in order to balance the COM (centre of Mass) of the body.

The advantage of the stereo-photogrammetric approach is that the postural variability induced by the position of the arms is completely avoided, thus allowing measurement of the true natural-neutral unconstrained erect-standing posture without any restriction. In such a posture, measurements of KTA, LTA and ST generated values and ranges (Table 3) are found to be very close to their X-ray based counterparts. Measured values were as follows: for KTA, μ = 0.65°±1.95°, range = -10.8° to 8.8°; for LTA, μ = -5.85°±2.34°, range = -16.8° to 10.8° [47,54]; and for ST, μ = -0.9°±3.1°, range = -7° to 5.2° [47,55]. As can be noted, offsets of 3° to 5° were found between these stereo-photogrammetric values and those reported in the literature, either in the average values or in the corresponding ranges. Such offsets found in the global or segmental spine tilts can be explained by reference to the different positioning of the arms, explained above.

It is interesting to note that a difference in KTA was found between genders. Males presented a more forward-tilted kyphosis than females even if the kyphotic curve was not statistically different between genders [21]. Conversely, LTA was equal between sexes while the lordotic curve showed statistically differing results [21].

The parameter that exhibits the most relevant offset by comparison to the X-ray based value (40°) [3,18] is S-SS, which was measured at approximately 16° (Table 3). This result emphasises that the angle between the sacral plate and the line connecting the S1 and S3 spinous processes is geometrically larger than the corresponding orthogonal angle which presents an additional offset of about 24°. All the spino-pelvic parameters which involve S-SS as a computational element are affected by this offset.

It is also important to note that the relationship between PI, SS and PT, as found in the X-ray based literature (see Vrtovec [18] for a review) still holds true for the stereo-photogrammetric approach in both computational methods described above; ie, PI = SS+PT when L5S1JC is used; and S-PI = S-SS+S-PT when the S1 marker is used. By geometric analogy a direct comparison with the X-ray based normative data can be performed only with the values obtained using the first method, while the second method represents the basis for future stereo-photogrammetric based studies and clinical application.

In the present study, significantly different PT values were found for females (15.3°, range 0.7°–29.6°) and males (17.3°, range 4.7°–30.1°). These values are fully compatible with the values reported in the X-ray based literature (12°, range 5°-30°) [3,49] where no gender difference has been documented. Interestingly no gender difference was found in the present study for the corresponding S-PT parameter. For this reason it could be argued that, as the L5S1JC position is derived from pelvic thickness and width, such a difference could reflect the gender difference for pelvis thickness as found in the study by Rajnics et al. [18,58]. The same consideration could hold true for the SPA values, as opposed to the S-SPA values, resulting from the statistical difference found to exist between sexes. By considering the offsets found for PT and SS values and their relationship (PI = SS+PT) it is easy to derive the difference found for the PI value (Table 3). By analysing the TPA and S-TPA parameters it becomes clear that in this case a difference between genders was found using both computational methods. For TPA the findings relating to L5S1JC and the differences in pelvic thickness could still be valid, but a greater influence might possibly be connected to the position of C7 with respect to CHA and/or (in ways yet to be confirmed) to the results for KTA showing males as having a greater forward kyphosis tilt than females.

The TPA numerical value found in our population appears to be fully equivalent to the 8.28°±5.82° reported by Yang et al. [59] where, unfortunately, no clear description has been given as to the position of the arms or the posture maintained during the X-ray data collection. Conversely, the TPA numerical value presents a 10° to 8° offset from that reported by Hasegawa et al (15.5°±8.6°) [56] with subjects standing with their hands placed on their cheeks. Regarding S-SSA, the found value in our study (103.7°±6.3°) is fully congruent with that presented by Mac-Thiong et al (130°±8.1°) [55] after taking into account the offsets of 24° found in S-SS and of about 3° in ST, due to the difference in standing posture maintained during data collection (see above).

Finally, considering the differences between genders found for the CZBA, showing males as having a more backward-tilted head as compared to females, it seems that a minor difference in local/global sagittal alignment strategies could be said to exist between the sexes. At the same time, upon reviewing the discussion above it appears that there was no difference between genders for S-SS, S-PT, ST, SVA and not even for LTA, although lordotic angle values were different between sexes [21]. Therefore, there might be grounds to conclude that males and females generate different local compensatory strategies in order to reach the same global alignment: as kyphosis is more backwards-tilted in females than in males, this might induce in the latter group a different head compensatory tilt in order to achieve a horizontal gaze. A possible biomechanical explanation of such female backward kyphosis tilt might relate to the forward biomechanical load created by breast mass; this could be an issue for future investigation.

### Limitations of the research

The measurement method/s used in this research contain three main sources of potential error: the accuracy of the 3D stereo-photogrammetric capture; operator-dependent skin marker positioning; and data processing. Furthermore, there are inherent limitations to the use of cutaneous markers, such as those used here, in different kinds of populations. These constraints will be addressed below.

In the first instance the study uses a well-established and easy-to-manage mature stereo-photogrammetric procedure [60,61]; moreover, present-day high-resolution stereo-photogrammetric systems provide very high 3D accuracy in 3D marker position reconstruction: 0.3–0.4mm range on a 3m x 3m x 2m working volume in the GOALS system used for the present study.

As regards the second issue, inaccurate and/or inconsistent marker positioning could be additional sources of potential error. This critical process must be performed with maximum accuracy because the measurements depend strictly upon the correct placement of the markers. In order to reduce marker misplacement, the authors use and recommend hemispheric markers that should always be positioned by palpation by an experienced operator while the subject is standing. Prior studies [62–68] have demonstrated via X-ray and Magnetic Resonance Imaging that skilled operators are able to perform accurate positioning by palpation with a relatively low rate of intra- and inter-examiner error. In one study [69] flaws were found in the measurement of frontal plane spine deformities/Cobb angles in scoliotic patients: the inaccuracy was caused by underestimation induced by marker misplacement. Despite this, the authors concluded that this caused no problems regarding the reproducibility of the sagittal spine profile using a marker-based approach; and specifically that absolute angular values in the sagittal plane curves could be measured with reasonable accuracy [69].

Third, data processing could be a primary source of data corruption if improper signal processing techniques are used. This is particularly important given that 3D spine shape and its related 1st and 2nd derivatives (necessary to proceed to an assessment of curve angles) must be identified using only 11 points [22,35,36]. Prior work by D’Amico et al. 1995 [22] originally introduced an efficient and specially devised numerical processing technique, showing a maximal error of less than 1° (approximately) for the Cobb computed angle on a curve of about 65°, modelled with a simulated sinusoidal data series with superimposed white noise (σ = 1mm).

In all cases, the likelihood of potential error from these sources has been conclusively demonstrated to be relatively small as compared to errors resulting from subject postural adjustment variability; additionally, it has been shown that such quantitative methods allow a higher degree of accuracy than the previous, alternative methods relied upon during clinical evaluations [64,65]. The normative 3D opto-electronic stereo-photogrammetric sagittal alignment parameters presented here are strongly consistent with the existing data obtained via X-ray based methods as found in the literature, for a healthy young population with controlled BMI. Both approaches aim to describe, and rely upon, the full-skeleton upright neutral standing attitude.

Nevertheless there are inherent limitations to the use of cutaneous markers (such as those used here) in different kinds of populations. For example, in cases where an individual has voluminous subcutaneous tissue the measurements might show a disparity with our results, particularly as regards the distance between the skin-marker and the reference bones on the spine; likewise, the surface profile might diverge from the actual shape of the spine or sacral bone sagittal tilt. The possibility of such practical conditions and variations should be fully taken into account in any future studies such as, for example, research and analysis of sagittal alignment in an elderly population that may present with a BMI range higher than the range found in the present study.

## Conclusions

In this study the authors have presented an innovative stereo-photogrammetry-based method of acquiring normative data for the purposes of global and spino-pelvic sagittal alignment. For spino-pelvic parameters two different approaches have been used. The first is intended to produce results as similar as possible to pre-existing X-ray based definitions, using two indirect measurements: ie, the assessed position of CHA [32] and an estimate of the location of L5S1JC [33,34]. The second approach avoids the L5S1JC assessment, considering in its place the position of a marker placed on the S1 spinous process. The values obtained for the first approach demonstrate a high level of agreement with X-ray based literature. The second set of parameters is not directly comparable with the X-ray based results but, given its reduced dependency on regression equations, has been developed as a reliable alternative set of reference values for future studies and clinical applications based on stereo-photogrammetric methodology.

The technique described in this study offers a number of advantages, specifically its non-invasive and innocuous qualities, the relatively short time needed for a rapid and automatic evaluation, the ability to provide a large number of clinically useful 3D and 2D anatomical/biomechanical/clinical parameters and, finally, the integration of these parameters into a comprehensive and coherent postural evaluation. These features will allow any future studies to carry out and directly compare multiple different postural examinations and/or assessments of sagittal alignment including sagittal spine shape variations occurring during gait performance, all obtained within a same-session time frame and in a statistically reliable way. The normative quantitative data presented here, based on a healthy young population, can serve as a reliable basis for future study of 3D posture and spine shape modification at any stage of life, from childhood to advanced age. In addition, this aggregated knowledge can provide an invaluable reference tool for the development of prevention strategies based on adapted physical activity programs.

In summary, the innovative non-ionising method described in this paper allows researchers and clinicians to measure, evaluate, intervene in, and monitor the treatment of their subjects so as to avert the risk of increasingly high ASD levels, especially prevalent as subjects advance in age over the course of a lifetime.

## Supporting information

S1 File Raw data source for normative 3D opto-electronic stereo-photogrammetric sagittal alignment parameters in young healthy adult population used in the statistical analysis (Data are provided as a MS-Excel data sheet file). (XLSX)
